# Mapping evidence on decision-making on contraceptive use among adolescents: a scoping review protocol

**DOI:** 10.1186/s13643-018-0881-8

**Published:** 2018-11-20

**Authors:** Mumbi Chola, Khumbulani Hlongwana, Themba G. Ginindza

**Affiliations:** 1 Discipline of Public Health Medicine, School of Nursing and Public Health, University of KwaZulu-Natal, Durban, 4041 South Africa; 2 Department of Epidemiology & Biostatistics, School of Public Health, University of Zambia, Lusaka, Zambia

**Keywords:** Adolescents, Decision making, Contraception, Contraceptive use

## Abstract

**Background:**

Contraceptive use among adolescents remains consistently low globally. Numerous studies have been done investigating factors that contribute to low contraceptive prevalence rates in this special population. It is particularly vital to understand decision-making processes that adolescents undergo when deciding whether or not to use contraceptives. Therefore, this scoping review seeks to map available evidence on decision-making processes in contraceptive use among adolescents.

**Methods:**

We will conduct a scoping review to explore, describe and map literature on the adolescent decision-making regarding contraceptive use. The primary search will include peer-reviewed and review articles. Databases, including PubMed, MEDLINE with Full Text via EBSCOhost, PsychINFO via EBSCOhost, CINAHL with Full Text via EBSCOhost, Google Scholar, Science Direct and Scopus, will be searched for articles that meet the eligibility criteria. Keyword searches will be used, and for articles included after title screening, abstract and full articles will be screened by two independent reviewers with a third as a decider on any disputes. Content analysis will be used to present the narrative account of the reviews.

**Discussion:**

Understanding how adolescents make decisions about whether or not to use contraception is essential for improving contraceptive prevalence rates in this special population. It is envisioned that the results from this review will highlight key evidence on how adolescent make decisions regarding contraceptive use as well as gaps and opportunities for future research. It will also be important in enhancing and re-focusing adolescent sexual and reproductive health policies and programmes.

## Background

Modern contraceptive methods are products or medical procedures that interfere with reproduction from acts of sexual intercourse [1]. Types of modern contraceptives include sterilisation (male and female), intrauterine devices and systems, subdermal implants, oral contraceptives, condoms (male and female), injectables, emergency contraceptive pills, patches, diaphragms and cervical caps, spermicidal agents (gels, foams, creams, suppositories, etc.), vaginal rings and sponge [1].

Globally, the prevalence of contraceptive use varies. In 2015, two out of three women or about 64% of women of reproductive age (15–49), married or in a union, were using some form of contraception, either modern or traditional [2]. Eastern and Southern Africa and West and Central Africa recorded lower numbers with 38.6% and 17.6% respectively [2]. Statistics are even lower among adolescents [3–5] with only about 15% of girls in developing countries aged 15–19, married or in a union, using modern contraceptive methods [6]. Adolescence is defined as all persons aged 10 to 19 years [7], and it is further subdivided into early adolescence (11 to 13 years), adolescence (14–17 years) and young adulthood (18–25 years), which encompasses those aged 18–19 years [8].

Low contraception use in this age group exposes adolescents to higher risks of maternal mortality, obstructed labour and obstetric fistula, and results in lower chances of receiving an education and obtaining employment [9–11]. Children born to adolescent mothers also face higher risks of mortality, undernourishment and school dropout compared to their peers [12].

While factors that contribute to low contraceptive use among adolescents have been well documented, it is particularly vital to understand decision-making process they undergo when deciding on whether or not to use contraceptives. This information will be vital for policy-makers and program managers in addressing poor contraception usage among adolescents as well as preventing maternal complications. It will also be important in enhancing and re-focusing adolescent sexual and reproductive health policies and programmes.

Therefore, this scoping review seeks to:

- Map existing literature on adolescent decision-making on contraceptive use in sub-Saharan Africa
- Map existing literature on the influence of parental, societal and peer-related factors on adolescents’ decision to use contraception

Findings from this review will highlight gaps in literature and form the basis for refining research questions for further research.

## Methods

### Scoping review

This is a scoping review of literature on adolescents’ decision-making on contraceptive use. This review is part of a larger study whose aim is to examine the levels, patterns and trends of contraception use among adolescents and understand their decision-making, as well as their needs, preferences and perspectives regarding existing and future contraceptive methods. The review has been written using the PRISMA-P [13] as a guide and will be based on the methodological framework for scoping studies as proposed by Arksey and O’Malley’s [14]. The framework stipulates the following steps:

- Identifying the research question
- Identifying relevant studies
- Study selection
- Charting the data
- Collating, summarising and reporting the results

This scoping review will, however, include a quality appraisal of the studies included in the review as proposed by Levac et al. [15].

### Identifying the research question

What is the available evidence on decision-making in contraceptive use among adolescents?

Sub-questions include:

1. What societal and peer factors influence adolescents’ decision-making on contraceptive use?
2. What parental factors influence adolescents’ decision-making on contraceptive use?
3. What individual factors influence adolescents’ decision-making on contraceptive use?

### Eligibility criteria

Eligibility criteria will be based on the following inclusion and exclusion criteria.

### Inclusion criteria

Studies present evidence on:

- Adolescent boys and girls aged 10–19 years
- Decision-making in contraceptive use among adolescents aged 10–19 years
- Parental influences on adolescents’ decision to use contraceptives
- Societal and peer influences on adolescents’ decision to use contraceptives
- Individual or “self” influence on adolescents’ decision to use contraceptives
- Published studies including guidelines, reports, technical or policy briefs and opinion papers and other grey literature

### Exclusion criteria

Studies meeting the following criteria will be excluded:

- Studies with no evidence on decision-making in contraceptive use among adolescents
- Studies with no evidence on influence of parental, societal, peer or individual factors on decision-making in contraceptive use among adolescents
- Studies not focused on adolescents aged 10–19 years
- Studies not freely available in full text

### Eligibility of research question

The study has used the Population–Concept–Context (PCC) framework (see Table 1) recommended by the Joanna Briggs Institute for scoping reviews [16] to determine the eligibility of research question. This is a more flexible alternative to the PICO (Population, Intervention, Comparator and Outcome) framework recommended for systematic reviews.

**Table 1:** PCC framework

Population	Adolescents aged 10–19 years. This is based on the World Health Organization (WHO)’s definition of adolescents [21].
Concept	Decision-making in contraceptive use. This includes factors that adolescents consider and processes that they go through in deciding whether or not to use contraceptives
Context	Global—including studies from high-income and LMICs

Identifying relevant studies (search strategy)

The following databases will be searched for articles that meet the eligibility criteria. These are PubMed, MEDLINE with Full Text via EBSCOhost, PsychINFO via EBSCOhost, CINAHL with Full Text via EBSCOhost, Google Scholar, Science Direct and Scopus. Search will follow the PRISMA guidelines

### Identifying relevant studies (search strategy)

The following databases will be searched for articles that meet the eligibility criteria. These are PubMed, MEDLINE with Full Text via EBSCOhost, PsychINFO via EBSCOhost, CINAHL with Full Text via EBSCOhost, Google Scholar, Science Direct and Scopus. Search will follow the PRISMA guidelines.

Articles will also be searched through the ‘Cited by’ search and reference lists of included articles. Search strategy was piloted to check the appropriateness of selected electronic databases and key words (Table 2). Boolean terms AND and OR will be used to separate the keywords during the search. Mesh terms (Medical Subject Headings) will also be included in the search.

**Table 2:** Keyword searches

Date searched	Keyword search terms	Search engine used	Number of studies
21-06-2018	((“adolescent” [MeSH Terms] OR “adolescent” [All Fields]) AND (“decision making” [MeSH Terms] OR (“decision” [All Fields] AND “making” [All Fields]) OR “decision making” [All Fields])) AND (“contraception” [MeSH Terms] OR “contraception” [All Fields])	PubMed	871
21-06-2018	Adolescent AND decision making AND Contraception	Via EBSCOhost	1752
		• MEDLINE with Full Text	653
		• PsychINFO	211
		• CINAHL with Full Text	108
21-06-2018	Adolescent AND decision making AND Contraception	Google Scholar	66,000
21-06-2018	adolescent AND decision AND making AND contraception AND (LIMIT-TO (ACCESSTYPE (OA)) OR LIMIT-TO (ACCESSTYPE (OTHER)))	Scopus	4836

**Fig. 1:**
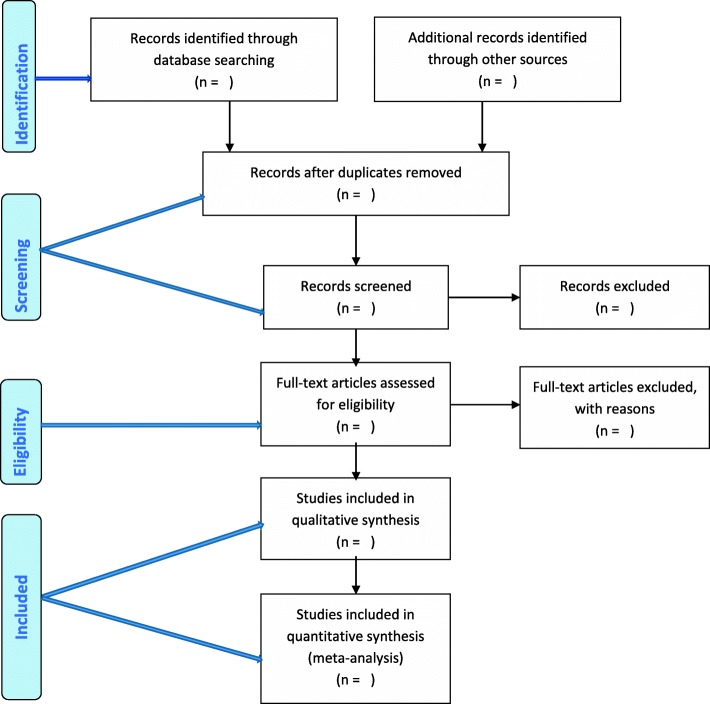
PRISMA flow diagram

A library will be created for this review using EndNote ×8.0.2 referencing software. The primary investigator will conduct a comprehensive search and screening of the study titles from the abovementioned databases. All studies with eligible titles will be exported to the EndNote library, and all duplicates will be removed before abstract screening. Two reviewers will independently conduct abstract screening followed by full-article screening of selected studies, using standardised tools, with guidance from the eligibility criteria. Where disputes arise, a third reviewer will decide. To optimise the article search strategy, we will utilise our local library services, the UKZN library services, to help with retrieving and finding articles to be included in the full-article screening. Where articles are unavailable, authors will also be contacted. Reporting on these will be done using the Preferred Reporting Items for Systematic Reviews and Meta-Analyses (PRISMA) chart [17], shown in Fig. 1.

### Data extraction/charting

A data charting table (Table 3) will be used to extract background information and process the information from each utilised study. The data extraction form will be developed, piloted and used to extract and process relevant information from each study included. All variables that will focus on answering the research question will be included. The research team will independently conduct a trial data extraction and later discuss as a group to determine consistency of the data extraction approach with the research question and objective. The data extraction form will be continually reviewed and updated in an iterative process. This will improve the quality applicability and consistency of the chart. Once this is completed, the primary author will conduct the data extraction which will be reviewed by the other authors. Any and all discrepancies will be discussed and agreed upon in the final interpretation. All articles reviewed will be assigned a unique code to help track all articles reviewed and those that will be excluded during the data charting process.

**Table 3:** Data extraction form

Author and date	
Study title	
Study population	
Gender	
Marital status	
Education level	
Methodology	
Geographical setting (country)	
Study site	
Study type	
Residence	
Study design	
Data collection methods	
Data collection tools	
Data collection method	
Sampling	
Sampling method	
Intervention (contraception)	
Contraceptive method	
Decision-making	
Data analysis	
Data analysis type	
Data analysis method	
Results	
Most important finding	
Other findings	
Conclusion	
Study limitations and recommendations	

### Collating, summarising and reporting the results

Once the data extraction is completed, a narrative account of the data extracted from the included studies will be analysed using the thematic content analysis. Data relating to adolescents’ decisions making in contraception and contraceptive use that will be extracted will be coded. NVIVO software version 10 [18] will be used collectively to code the data from the included studies. Emerging themes will be identified and data will be coded according to these themes. In line with the general aim of a scoping review, to map out the research landscape, some form of visual representation of the data will be presented in the results section to map the extent, range and nature of research in this area. This will help to identify patterns and themes and postulate explanations for summarising and synthesis of findings. The process will be done as follows [19]:

- Coding data from the included articles
- Categorising the codes into major themes
- Displaying the data
- Identifying key patterns in the data and identify sub-themes
- Summarising and synthesising

Resulting themes will then be analysed and synthesised, and their relationship to the research question and objective will be critically examined. The meanings of the findings in relation to the aim of the study and the implications of these findings for future research, policy and practice will be examined.

### Quality appraisal

The Mixed Method Appraisal Tool (MMAT)-version 2011 [20] will be used to determine quality of the studies. Depending on the study design, the appropriate section will be used. Section 1 will be used to appraise qualitative studies; sections 2 to 4 for quantitative studies and section 5 for mixed methods studies. The MMAT will be used to examine the appropriateness of the aim of study, adequacy of methodology, study design, data collection, study selection, data analysis, presentation of findings, author’s discussions and conclusions. The scoring matrix in the tool will be used to grade the overall quality. The results from scoring of the abovementioned aspects will determine the quality of resultant article.

## Discussion

Understanding how adolescents make decisions about whether or not to use contraception is essential for improving contraceptive prevalence rates in this special population. Increasing contraception among adolescents is important because it will help prevent adverse health such as maternal mortality, obstructed labour and obstetric fistula, and socio-economic outcomes such as diminished opportunities for education and employment [9–11]. Conducting this systematic scoping review will map and document existing evidence on factors that adolescents consider and decision-making processes they go through in deciding whether or not to use contraceptives. This information is vital for understanding why contraceptive prevalence rates among adolescents remain consistently low.

This systematic scoping review will focus on studies published between 1990 and 2017. This is because during this period, there have been various programmes and projects aimed at improving contraception among adolescents in Zambia. The focus is on adolescents because they have been identified as a special population whose health needs have to be prioritised. It is envisioned that the results from this review will highlight key evidence on how adolescent make decisions regarding contraceptive use as well as gaps and opportunities for future research.

## Abbreviations

LMIC: Low-to-middle-income country
MMAT: Mixed Method Appraisal Tool
PCC: Population–Concept–Context
PICO: Population, Intervention, Comparator and Outcome
PRISMA: Preferred Reporting Items for Systematic Reviews and Meta-Analyses
UKZN: University of KwaZulu-Natal
WHO: World Health Organization
